# Identification and Antibiotic-Susceptibility Profiling of Infectious Bacterial Agents: A Review of Current and Future Trends

**DOI:** 10.1002/biot.201700750

**Published:** 2018-08-26

**Authors:** Gaetano Maugeri, Iana Lychko, Rita Sobral, Ana C. A. Roque

**Affiliations:** UCIBIO, Departamento de Química, Faculdade de Ciências e Tecnologia, Universidade NOVA de Lisboa, 2819-516 Caparica, Portugal; UCIBIO, Departamento de Ciências da Vida, Faculdade de Ciêcias e Tecnologia, Universidade NOVA de Lisboa, 2819-516 Caparica, Portugal; UCIBIO, Departamento de Química, Faculdade de Ciências e Tecnologia, Universidade NOVA de Lisboa, 2819-516 Caparica, Portugal

**Keywords:** antimicrobial resistance, antimicrobial susceptibility profiling, bacteria identification, clinical microbiology, diagnostic methods, infectious diseases

## Abstract

Antimicrobial resistance is one of the most worrying threats to humankind with extremely high healthcare costs associated. The current technologies used in clinical microbiology to identify the bacterial agent and profile antimicrobial susceptibility are time-consuming and frequently expensive. As a result, physicians prescribe empirical antimicrobial therapies. This scenario is often the cause of therapeutic failures, causing higher mortality rates and healthcare costs, as well as the emergence and spread of antibiotic resistant bacteria. As such, new technologies for rapid identification of the pathogen and antimicrobial susceptibility testing are needed. This review summarizes the current technologies, and the promising emerging and future alternatives for the identification and profiling of antimicrobial resistance bacterial agents, which are expected to revolutionize the field of clinical diagnostics.

## 1 Introduction

By discovering penicillin in 1928, Sir Alexander Fleming triggered the beginning of the modern era of antibiotics, which revolutionized medicine and society, saved lives, and increased the life expectancy to what we know today. The remarkable effectiveness of antibiotics led to the euphoria mistaken belief that all infectious diseases could be successfully controlled with antibiotics. However, during the past few decades, the imprudent and excessive use (underuse, overuse, and misuse) of antibiotics regrettably led to the rapid emergence and propagation of bacterial strains resistant to virtually all therapeutically useful antibiotics.[1] The increasing frequency of infections by antimicrobial-resistant bacteria is due to their capacity to recurrently develop new mechanisms of resistance. The lack of alternative treatments results in longer hospital stays, delayed recovery, long-term disability, and an increase in public healthcare costs. In the USA, the estimated healthcare cost associated to antimicrobial resistance (AMR) was $55 billion per year in 2013, and 2 million people were sick every year due to antibiotic-resistant infections, with over 23 000 deaths as a result.[2] In Europe, the 2009 report from European Centre for Disease Prevention and control (ECDC) and European Medicines Agency (EMEA)[3] estimated overall societal costs over 1.5 billion € per year, with over 900 million € in hospital costs. In the EU, about 25 000 patients died due to multidrug-resistant (MDR) bacteria infections.[3,4] It should be noted that the emergence and spread of AMR bacteria are prevalent in both healthcare and community settings, typically known as healthcare-associated infections and community-acquired infections.

The World Health Organization (WHO) recently published a priority list of antibiotic-resistant pathogens. Gram-negative carbapenem-resistant *Acinetobacter baumannii*, carbapenem-resistant *Pseudomonas aeruginosa* and carbapenem-resistant and third-generation cephalosporin-resistant Enterobacteriaceae are at the top of this list, classified as critical priority agents. In the high-priority list, gram-positive bacteria for which there are treatment options likely to be successful, were included, namely the methicillin-resistant, vancomycin-intermediate and -resistant *Staphylococcus aureus* and the vancomycin-resistant *Enterococcus faecium*.[5] The list does not include *Mycobacterium tuberculosis*, as it is a globally established priority, urgently needing innovative treatments, and already targeted by several dedicated programs. The WHO[6] also suggested that global research should focus on the development of new diagnostic and therapeutic tools.[7]

**Figure 1:**
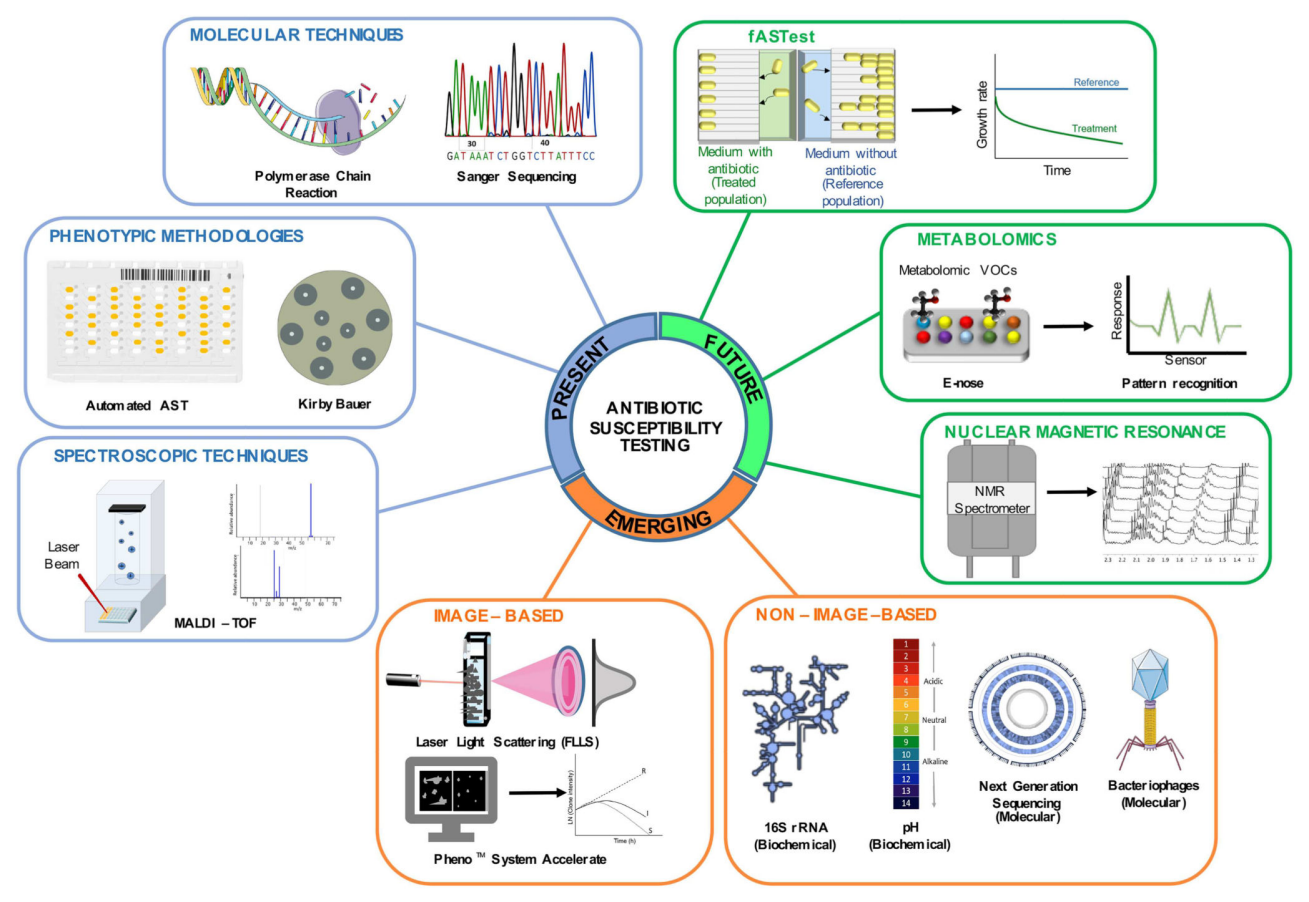
Summary of current, emerging, and future technologies for the identification of bacterial pathogens and for antimicrobial susceptibility testing in clinical diagnostics of infectious diseases. Current technologies are considered those that are nowadays in use in clinical settings, certified, and commercially available; emerging technologies are those entering the market and reaching regulatory approval; future technologies are those under development.

The current review aims at presenting the current, emerging, and future technologies implemented or in development, which target the early identification of the pathogenic agent as well as a fast antibiotic susceptibility profiling. An overview of the different methodologies is summarized in Figure 1.

## 2 Current Technologies for Bacterial Identification and Antibiotic Profiling

**Figure 2:**
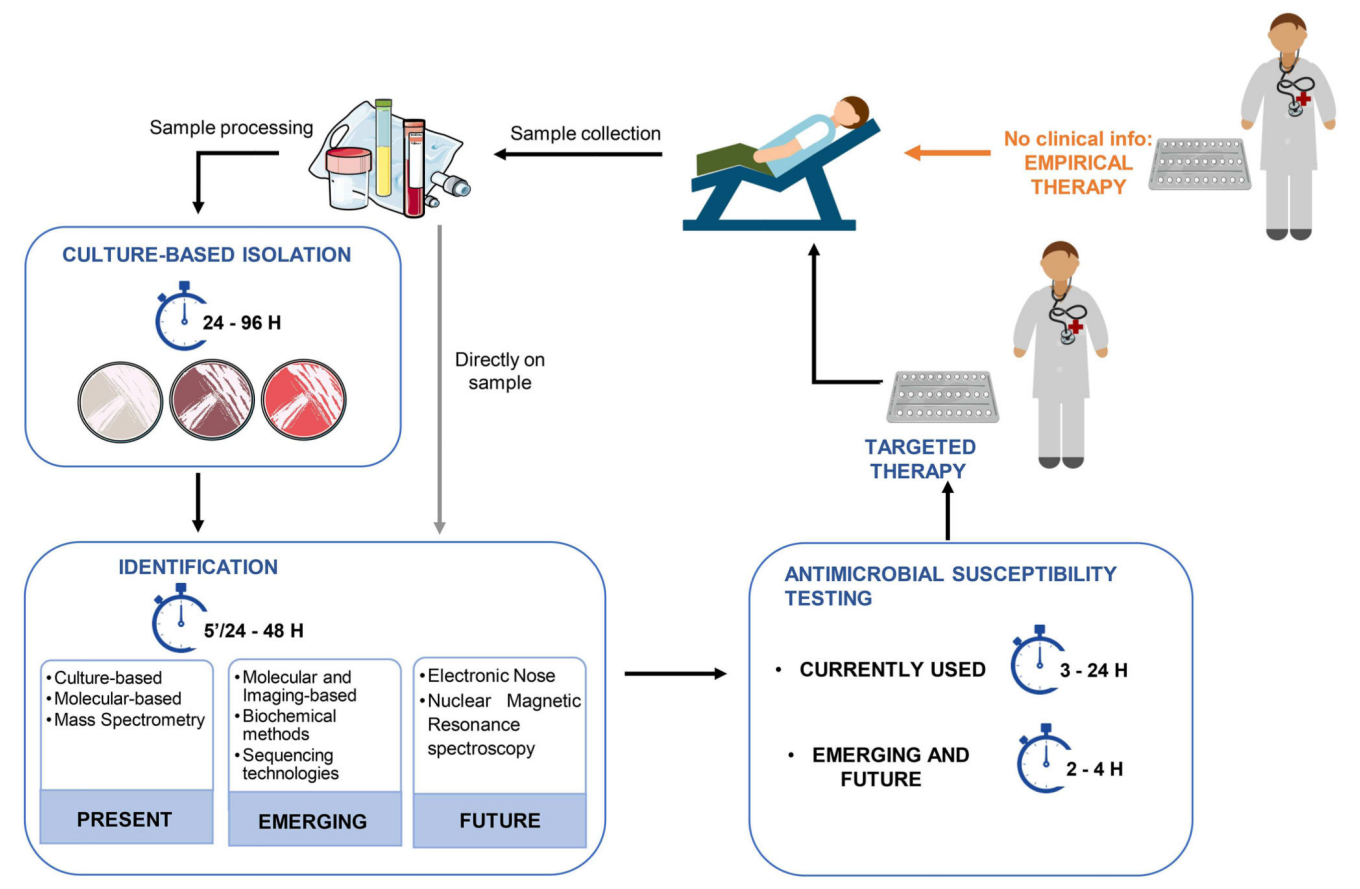
Typical procedures currently in place in clinical settings to provide identification of the pathogen agent and the profiling of antimicrobial susceptibility.

A clinical microbiologist has usually two main goals when processing clinical samples. One goal is to isolate and identify the pathogen causing the infection. A second goal is to evaluate its antibiotic susceptibility profile, providing useful information to prescribe the most efficient antibiotic treatment. The typical workflow currently in place for pathogen identification and antimicrobial susceptibility testing (AST) is presented in Figure 2, and the methodologies used are summarized in Table 1.

The isolation of pathogens from clinical samples still occurs through culture methods, using agar-based media (nutritive, differential, and/or selective). Some clinical laboratories use chromogenic agar-media harboring chromogenic or fluorogenic substrates that are hydrolyzed in the presence of specific enzymes. Several tests are then performed to address genus identification, namely microscopy cell staining, colony morphology, and rapid biochemical tests. To perform identification at the species level, the more common methods are phenotypically based, such as manual (e.g., Api bioMérieux) and automated biochemical tests, which exploit the differences in protein expression within genus (or also between genera), providing a characteristic protein expression fingerprint with a relatively high degree of certainty.[8] For example, the OmniLog ID system (Biolog) is a rapid method for the phenotypic identification of bacteria and fungi, through their ability to oxidize different carbon sources. Here, each well of the card contains one of 94 different carbon compounds and a tetrazolium-redox dye, used as a flag to indicate if the microorganism tested has or not utilized the carbon compound, providing a “metabolic fingerprint” of the microorganism.[9] Although useful and easy to operate, agar-based media and biochemical tests are not completely specific and occasionally fail or provide presumptive identification (percentage of possibilities). Therefore, further confirmation of species identity is often required. Different approaches, not culture-based, either current and emergent can be used, some of these are able to provide both identification and antimicrobial-susceptibility data simultaneously.

Despite the introduction of new time-saving technologies to obtain antimicrobial-susceptibility data, the current (classic) technologies are still used in many hospitals. These include culture-based, molecular-based and, more recently, spectrometry-based approaches or molecular and microscopy-based approaches. The first rely on the detection of phenotypic resistance by determining bacterial growth in the presence of antibiotics. These can be divided in manually performed tests (agar dilution, disk diffusion, gradient test, and broth microdilution) and automated systems. All these technologies provide qualitative data (e.g., susceptible, intermediate, resistant) for the strain tested and, except for the disk diffusion, also provide the quantitative values of the MIC (minimum inhibitory concentration), defined as the lowest antibiotic concentration that inhibits the visible growth of an organism after overnight incubation.[10] The interpretative standards for these classifications are reviewed and updated annually by several organizations such as the Clinical and Laboratory Standards Institute (CLSI)[11] in the USA and the European Committee on Antimicrobial Susceptibility Testing (EUCAST).[12]

### 2.1 Culture-Based Techniques

The dilution assay can be done in agar (agar dilution) or broth medium (macro- or micro-dilution). The agar dilution assay is performed in several Petri dishes of Mueller-Hinton agar (MHA) supplemented with the appropriate dilutions of antibiotic, each plate containing a different concentration. Antibiotic-free plates are used as control. Despite its laborious set up and the short shelf life of the plates, this method has the advantage to simultaneously test up to 36 different inocula in the same plate.[10,13,14] The micro-dilution technique has been miniaturized using 96-well plates to test about 12 different drugs using a wide range of eight twofold serial dilutions in a tray.[15] After overnight incubation, development of turbidity or sediment in the wells indicate growth of the organism and the MIC values can be evaluated following the CLSI or EUCAST breakpoints.[4]

In the disk diffusion assay, the surface of the MHA plate is inoculated with a standardized inoculum of the test microorganism. Commercial filter paper disks impregnated with antibiotics at predetermined concentrations are placed on the agar surface and the antibiotic concentrations are directly reversed to the distance from the disk.[15,16] The disk diffusion assay provides only qualitative results (e.g., susceptible, intermediate, resistant), obtained by measuring the diameter (mm) of bacterial growth inhibition around the disk. Such data can be insufficient as it does not provide the physician with the MIC value, that may be needed for an efficient prescription.[10]

The gradient test meets the advantages of simple handling and the simultaneous use of several drugs as in the disk diffusion assay, while allowing MIC value determination as in dilution assays. Different commercial versions are available as *E*-Tests (BioMérieux) or MIC Test Strip (Liofilchem) using a similar procedure: the strip is impregnated with a gradient of predefined concentrations of the antibiotic, within the dilution range used in conventional methods for MIC determination.[15] Following inoculation on the surface of the MHA plate, with a standardized suspension of the microorganism, the plate is incubated overnight to allow bacterial growth and eventual drug-inhibition. The graduated scale on the antibiotic strip enables the reading of MIC which is determined by the intersection of the lower part of the ellipse shaped growth inhibition area with the test strip.[14] This methodology allows the simultaneous use of more than one strip. Although the cost of each strip is low (around 2–3 €), this methodology becomes expensive if several drugs have to be tested.[14] The fractional inhibitory concentration index (FICI) usually used to investigate if the antimicrobial interaction between two drugs are additive, synergistic, or antagonistic, can also be determined.[17,18]

Automated methodologies allow both identification and AST determination. These systems involve the use of cassettes (also called panels or cards) with a series of wells, each containing an individual substrate for pathogen identification. The metabolic activity of the strain such as acidification, alkalinization, and enzymatic hydrolysis of different substrates is analyzed.[19] The AST is based on the automation of the broth microdilution assay through sensitive optical detection systems, that measure bacterial growth in the presence of antibiotics, within 24 h post incubation.[20] The number and concentration of antibiotics tested is limited and their sensitivity is low, as a high number of viable cells is required to measure bacterial growth and turbidity changes. Other relevant weaknesses include the impossibility to process directly patient samples,[20] the absolute need of a pure culture of the pathogen, and the long processing time (several hours for identification and up to 18 h for AST).[21] The major advantages are their high degree of standardization according to the international guidelines criteria of CLSI and/or EUCAST,[22] and their capacity to manage high workloads. In fact, automated systems are commonly used in clinical microbiology laboratories which process a large number of daily tests.[22,23]

### 2.2 Molecular-Based Methodologies

Most of the molecular-based technologies rely on conventional polymerase chain reaction (PCR) or quantitative real-time PCR (qRT-PCR), to amplify specific sequences of nucleic acids, allowing simultaneous pathogen identification and AST.[24] However, it requires a DNA extraction step from isolated strains, a high number of cells to obtain sufficient DNA and previous knowledge on the sequences to amplify.[24] For some technologies, the possibility to identify the pathogen directly from the patient sample, namely whole-blood, serum, blood culture, or urine, clearly represents an advantage.[25–27] In fact, PCR-based techniques can be separated into culture-independent PCR assays, performed directly on raw samples, and culture-enriched assays, which instead require a previous growing step of the raw sample in an enrichment media.[28]

In the case of suspicion of bloodstream infections (BSI) or sepsis, molecular-based tests are essential for successful therapy choice, since they provide bacterial identification and/or detection of resistance traits, directly on the patient’s sample. For further details on rapid molecular diagnostics for BSI please read recent works.[25,29,30] We shortly describe the SeptiFast test (Roche), a culture-independent amplification assay, as it is the most studied and validated assay for the diagnosis of BSI. It performs bacterial identification directly on EDTA blood-whole samples (so it can be used in suspected bacteremia), and can detect 25 clinically relevant bacteria and fungi in about 6 h,[31–33] with a sensitivity of 3–30 CFU mL^−1^.[34] The assay uses dual FRET probes with two different fluorophores allowing quantification besides identification.[35] The technology using the FRET probe assay is restricted to the LightCycler instrument (Roche), and to the High-Resolution Melting (HRM) assay, requiring highly advanced qRT-PCR instruments.[36] Several studies evaluating the SeptiFast technology, reported sensitivity values in the range of 60–95% and specificity values of 74–99%, depending on the target pathogen. However, it is labor-intensive, needs professional expertise, and has a high associated cost (about 200 € per test). Moreover, except for methicillin-resistant *S. aureus* (MRSA), it provides no information on antimicrobial susceptibility.[29] SeptiFast can be used as a complement for traditional culture-based methods, in antibiotic-treated patients, as recently shown.[37]

Another culture-independent assay using qRT-PCR, approved by the US FDA, is the Gene Xpert system (Cepheid). It can detect MRSA and methicillin susceptible *Staphylococcus aureus* (MSSA) based on sequences of *spa*, *SCCmec,* and *mecA* genes with a turnaround time of 1 h from positive blood cultures.[29] The reported sensitivity for *S. aureus* detection is 100%, and 99.4% specificity for MRSA detection.[38–40] However, methicillin-resistant coagulase-negative *Staphylococcus* spp (CoNS) can lead to false positives in the *SCCmec* gene test.[29]

PCR-based technologies can also be used to identify ESβL and carbapenemase resistance genes in the Enterobacteriaceae family. The commercially available Line Probe Assay (LPA), uses conventional PCR followed by reverse hybridization of PCR amplicons, and simultaneously detects the presence of these two characteristic resistance genes in gram-negative bacteria.[41] A commercial example is the AID carbapenemase LPA (Auto-immun Diagnostika), CE-cleared, evaluated both in culture-independent assays (urine samples) and in DNA samples extracted from enriched bacterial cultures, for the detection of a wide list of carbapenem-resistant genes. The test has 100% sensitivity and 100% specificity when used in clinical isolates.[42] Although these tests detect the determinants of resistance, they have an important limitation: the presence of these resistance genes is not always correlated with phenotypic resistance.[24] This occurs in gram-negative bacteria, for which the resistance gene may be present, but at very low expression levels. Changes in the expression level occur through the insertion of mobile insertion sequences, which can act as better promoter regions, enhancing expression.[43] In gram-positive bacteria, the correlation between the genotype and the resistance phenotype is more reliable.[24]

Finally, qRT-PCR can be used for AST. It quantifies DNA copies and can detect bacterial growth in the presence of different antibiotic concentrations, being used to differentiate susceptible from resistant strains.[24] An example is the recently used test for antimicrobial susceptibility of clinical isolates of *A. baumannii* to imipenem, ciprofloxacin, and colistin in about 6 h (from isolated colonies). The bacterial growth was indirectly established through the detection of a highly conserved region of *ompA* gene.[44] Failure to limit the analysis to viable cells is the main limitation, as the presence of non-viable bacteria can overestimate the number of cells present in the sample and lead to increased false positive rates.[45]

For culture-independent assays, elimination of the enrichment step ensures a shortened time to result, bypassing the need for detectable bacterial growth. However, in antibiotic-treated patients, negative blood cultures can lead to false negative results, masking an occurring BSI or sepsis infection. Also, the low volume of blood used in the amplification step and consequently, the low nucleic acid contents, may not be enough to reach clinically relevant sensitivity.[46] To provide timelier results, the FDA- and CE-cleared FilmArray (BioFire) Blood Culture Identification (BCID) panel is used directly on positive blood culture bottles with a turnaround time of about 1 h. A list of 24 etiologic agents of sepsis is screened, including 8 gram-positive and 11 gram-negative bacteria, five yeast (*Candida* spp.) and three resistance genes, *mecA, vanA/B,* and *KPC*.[47] The BCID panel also detects contaminant bacteria. The assay consists of several automated steps, first cell lysis, nucleic acid purification, multiplex PCR, nested PCR and finally amplicon melt analysis. In the two-stage PCR, a multiplex step provides nucleic acid amplification for the subsequent nested PCR reactions. These reactions occur in an array, each well containing specific internal primers for the resistance and species marker genes. The real-time detection of multiple gene targets is achieved using a fluorescent double-stranded DNA-binding dye. The combinatorial result of the signals from the different wells gives the final result.[47,48] The overall sensitivity of the test ranges between 50% and 100%, while the overall specificity is reported in the range 77–100%.[49–51]

FilmArray has recently been compared to Verigene (Nanosphere, USA). Although these two FDA-cleared assays rely on completely different technologies, they both perform multiplex identification of individual targets.[52,53] Verigene includes two panels for bacterial identification, BC-GN that detects eight gram-negative bacterial targets and six key resistance markers, and BC-GP that detects twelve gram-positive targets and three associated resistance markers (the same as FilmArray), but lacks a panel for yeast detection. Concerning detection of resistance genes, Verigene can identify four carbapenemase (VIM, IMP, OXA, and NDM) and an ESBL gene (CTX-M).[52,53] The hands-on time is comparable for both systems, with a turnaround time of 2.5 h for Verigene. The Verigenes’s cartridges consist of a glass slide (microarray) and the associated technology is able to identify and quantify nucleic acid sequences, without an initial PCR amplification step. Several capture oligonucleotides probes, designed for a specific DNA sequence of the target pathogen, are printed over the microarray slide. Mediator oligonucleotide probes, containing a polyA tail, bind specifically to a second DNA region of the target pathogen and then to a poly T probe associated to a gold nanoparticle.[53–55] Subsequently, silver particles are deposited around the gold nanoparticles and their localization is detected by light scattering, providing accurate detection of the target sequences captured on the array.[54] The BC-GP showed values of 92.6–100% and of 95.4–100% for sensitivity and specificity of identification, respectively, and 98.6–100% and 94.3–100% for detection of resistance markers.[56] The BC-GN showed a sensitivity of 97.1% and a specificity of 99.5% for gram-negative bacteria.[57]

### 2.3 Spectrometry MALDI-TOF MS

**Figure 3:**
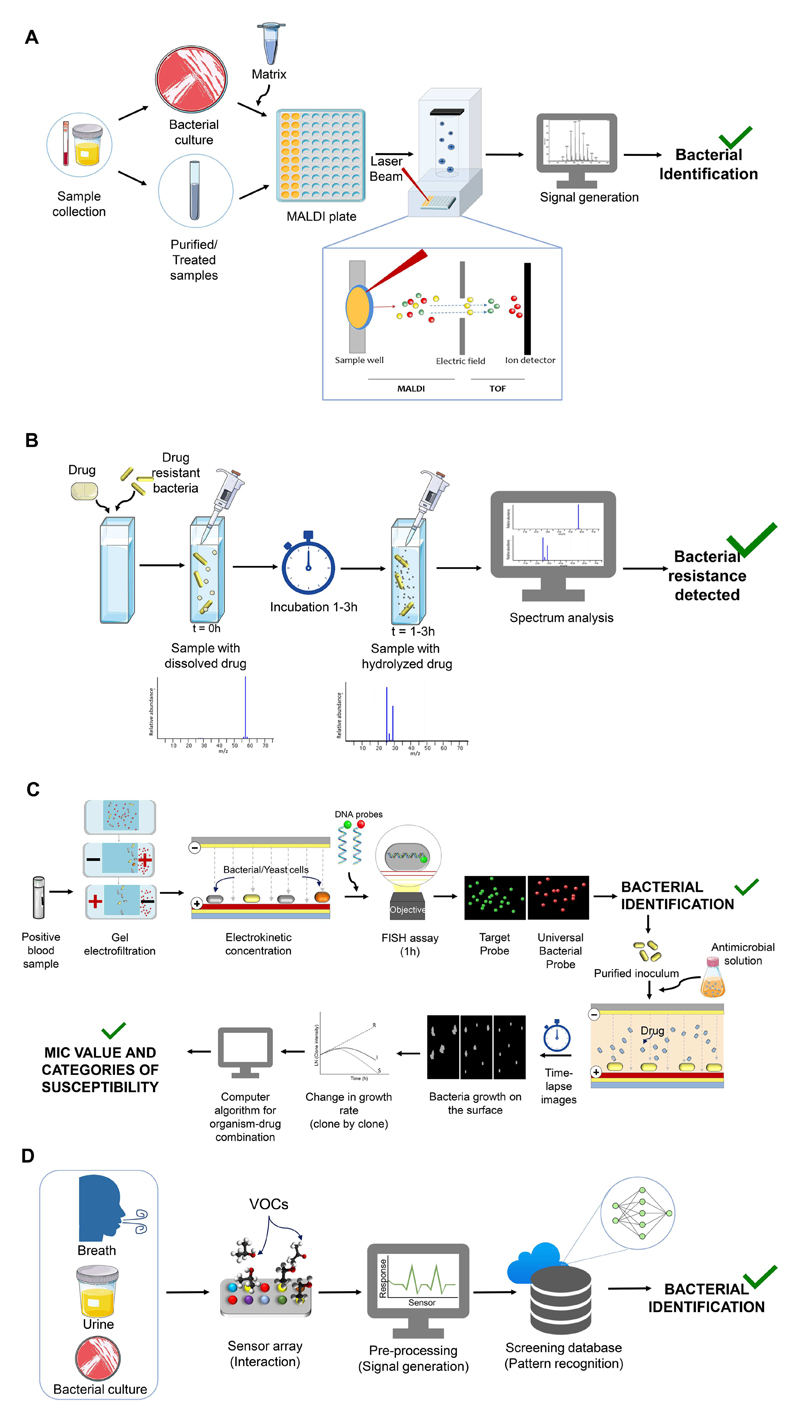
Detailed representation of the operation mode of technologies for pathogen identification and antimicrobial susceptibility testing. The use of MALDI-TOF MS for the identification of microbial pathogens A) and for antimicrobial susceptibility profiling B). Schematic workflow of Accelerate Pheno System for identification and antimicrobial susceptibility testing from positive blood cultures C). How Electronic Noses can profile bacterial volatile organic compounds D).

MALDI-TOF MS is based on the rapid ionization of the bacteria/yeast ribosomal proteins using a laser pulse, directly from cultured colonies or cell pellets from the clinical sample (Figure 3A). The calculated mass of the ions is the specific sample fingerprint of the bacterial/yeast species. This technique is nowadays widely used in clinical microbiology laboratories, particularly at University Hospitals.[58]

MALDI-TOF MS can be used for gram-positive, gram-negative bacteria and yeast, and does not require a specific test, in contrast to biochemical differentiation methods. However, similarly to these, it requires fresh colonies (not more than 48 h), as peaks become more difficult to distinguish, probably due to ribosomal protein degradation. The cost of each test is low, 1 € or less per sample, but a typical MALDI-TOF MS system costs €180 000–200 000, including analysis equipment, hardware, relevant software, and integrated databases.[58] MALDI-TOF MS workflow is high, processing 16–384 samples in a typical plate. Each sample analysis takes about 5–7 min, so the results are usually available 12–24 h after receiving the sample. The amount of cells required is low (10^4^–10^6^ CFU), and theoretically it can be performed using a single colony, obtained in few hours, from the culture of an infected sample.[58] Even if the identification at species level is possible it does not differentiate species with similar ribosomal protein sequences (*Shigella* spp. and *Escherichia coli* or *Streptococcus pneumoniae* and members of the *Streptococcus oralis*/*mitis* group); in these cases, other assays such as classical biochemical tests, antigen detection or molecular methods, are required. For the analysis of yeast with strong cell wall, a short extraction procedure may be required to provide the ribosomal proteins available for analysis.[58]

Some studies describe MALDI-TOF MS as a possible alternative technology for AST,[58] by directly analyzing the enzymatic reaction at the molecular level (Figure 3B).[59] The carbapenemase activity in gram-negative bacteria was evaluated by incubation of the bacterial samples (carbapenemase-carrying or non-carrying strains) with Ertapenem (carbapenem) and subsequent analysis through MALDI-TOF MS. Different mass peaks were obtained at the beginning of incubation and after 2.5 h, the time needed to evaluate the hydrolysis of β-lactam rings by the carbapenemase-carrying strain. Remarkably, the bacterial strains producers of NDM-1 or IMP-1 enzymes, were detected in just 1 h, the time to complete β-lactam hydrolysis.[60] Other carbapenems were evaluated, for instance Meropenem showed 96.67% sensitivity and 97.87% specificity values for *P. aeruginosa* and Enterobacteriaceae strains,[61] and Imipenem showed 100% sensitivity and specificity for *A. baumannii* strains.[62] A similar approach was used to screen other β-lactam substrates, to evaluate the possibility to distinguish other β-lactamases such as AmpC or TEM-1.[63] However, as for PCR-based technologies, MALDI-TOF MS may not provide a direct correlation between the presence of the hydrolytic enzymes and the phenotype of resistance[24] since some mechanisms, such as alterations in porins or upregulation of efflux pumps, are not detected.[59] In gram-positive bacteria, MALDI-TOF MS can be used to discriminate between *E. faecium vanB* positive and negative strains.[64] However, discrimination between MSSA and MRSA strains is contradictory, as some authors described measurable differences in the spectra,[65,66] while others considered such differences attributable to clonality.[67,68]

The difficulty in using MALDI-TOF MS directly on a clinical sample is due to the high amount of host proteins, This problem can be minimized by applying time consuming sample preparation methods.[58] Some clinical samples, such as urine, are easier to be directly processed, as they do not contain commensal flora nor host proteins, expected in some pathological conditions. In fact, pathogens were correctly identified at the species-level directly from urine samples at rates of 91.8% using a specific fast protocol.[69] However, the best results are obtained for high bacterial counts (>10^5^ CFU mL^−1^) and for gram-negative bacteria. A reliable protein profile is obtained only for bacterial counts of at least 8 × 10^4^ CFU mL^−1^ that corresponds to the diagnostic threshold for most UTIs. Finally, this method can under evaluate some UTIs like cystitis, which emerge with lower bacterial counts and lead to false negative results in these cases.

### 2.4 Spectrometry Approaches Combined with Molecular Tools

The polymerase chain reaction/electrospray-mass spectrometry (PCR/ESI-MS) is a fairly recent technology that couples a molecular method to a spectrometry approach, overcoming weaknesses in the analysis of complex samples and performing culture-independent analysis. It was originally developed by Ibis technology for biodefense and public health safety purposes, enabling rapid detection and identification of pathogens in clinical and environmental samples.[70] Later, Abbott Molecular acquired Ibis technology[71] and designed a more robust model, PLEXID[70] that was recently improved to IRIDICA, the newest PCR/ESI-MS system.[72] IRIDICA was evaluated in complex samples, such as whole blood,[73] bronchoalveolar lavage (BAL) [72] and in the diagnosis of endocarditis,[74] obtaining the CE marked designation in 2015.

The IRIDICA BAC BSI assay, for the evaluation of BSI and sepsis, includes a pre-filled 16-well PCR strip, with 18 primer pairs that target broadly conserved bacterial and fungal sequences of pathogenic species and also specific antibiotic resistance markers like *mecA*, *vanA*, *vanB*, and *blaKPC*.[73] Subsequently, the amplicons are submitted to ESI-MS. The system can detect over 780 bacterial and candida species using a proprietary database and software that compares the DNA sample sequence with a sequence library. The limit of detection ranges between 0.25–128 CFU mL^−1^, depending on the target species and the estimated time to result is 5 h and 55 min. The costs of the IRIDICA system and for each test are £268 000 and £174, respectively.[75] Four studies comparing the IRIDICA BAC BSI, with blood culture as reference method, reported an estimated summary specificity of 0.84 and sensitivity of 0.81.[75]

## 3 Emerging Technologies for Bacterial Identification and Antibiotic Profiling

Some recently emerging technologies rely on the measurement of several phenotypic features such as growth, morphology, viability and metabolism, using a wide range of different approaches (sometimes concerted) for fast identification and AST such as imaging-based, non-imaging-based, molecular-based, and biochemically-based (Table 2).

### 3.1 Imaging-Based Technologies

The image-based assays that can provide pathogen ID and AST, represent an impressive emerging technology in the field of clinical microbiology. Among these tools, the oCelloScope (Philips BioCell) performs AST, by relying on real-time analysis using an automated optical detection system, which analyzes up to 96 combinations of samples. The digital time-lapse microscopy scanning through the fluid sample generates a series of images. The optical resolution is comparable to a 200× magnification in a standard light microscope. Two algorithms, based on either pixel histogram summation or contrast segmentation and extraction of surface area, determine the bacterial growth kinetics, through image stack processing. This system was evaluated in several experiments, such as monoculture infection, with results obtained in 6 min for *E. coli* isolates and in 30 min for complex samples such as urine collected from pigs with catheter-associated UTIs.[65] It was also evaluated in positive blood cultures and the average time to obtain susceptibility degree values ranged from 1 to 4.2 h depending on the bacteria–antibiotic combination.[66] In contrast to competitor systems, the oCelloScope does not analyze single cells, but populations, and has lower resolution imaging. However, it has the advantage to allow bacterial growth measurement, without the need to attach the bacterial cells to an inert surface, a step that is required by other tools.[20]

In other higher resolution tools, multichannel test cassettes are used for real-time observation with high-resolution cameras, allowing the direct observation and measurement of bacterial growth. An example of such technology is the multiplexed automated digital microscopy (MADM).[76] A commercially available device using this technology is the Accelerate Pheno System (Accelerate Diagnostics) that can perform both identification and AST of bacteria and yeast and allows the diagnosis of mono and polymicrobial infections directly from blood-cultures, dismissing the overnight sub-culturing step. This technology uses two different approaches to achieve identification and AST, the first is obtained within 1.5 h and the latter within 6.5 h, and both occur inside the flow cells of a multichannel test cassette. The blood culture follows a series of automated processes (Figure 3C), starting with gel electrofiltration, that separates impurities, such as lysed blood cells and debris, from bacterial or yeast cells; the second step involves cell immobilization via electrokinetic concentration, which enables the microscopy-based single-cell analysis to achieve identification. The process of identification is performed through hybridization in situ with specific fluorescent probes (FISH) for bacterial and yeast cells, and with universal probes to obtain quantification and resolve the possible polymicrobial sample. The identification drives automatically the choice of antibiotics to be used. The sample is subjected to a pre-growth step to normalize growth rates, and a universal nucleic acid stain is used to quantify the number of cells in the purified inoculum. The flow cells are filled with an appropriate volume of purified inoculum and subsequently injected with MHA to perform the AST analysis. The susceptible cells are killed or their growth is inhibited by the treatment. Subsequently, using algorithms and mathematical regressions, based on the response of isolates with known MICs for a given antimicrobial, the growth curves are converted into MIC values.[77]

A study of BSI by gram-negative bacteria, demonstrated its capacity to correctly identify 88.7% of all episodes from blood cultures, including 10 polymicrobial BSI. However, in seven of the polymicrobial samples, cultivated gram-positive organisms were not detected.[78] This device was evaluated to successfully discriminate MRSA/MSSA, clindamycin resistance/susceptibility and VSSA/hVISA/VISA respectively using cefoxitin, erythromycin, and vancomycin.[79] It was also able to evaluate the processes of induction and heteroresistance, observing 10 or more growing clones per test, changing the time of exposition and the drug concentration. In only 3 h, the microscopy analysis succeeded to discriminate MRSA from MSSA, with a time gain of 15 h in comparison to the microdilution method and of 44 h to differentiate between VSSA, hVISA, and VISA in comparison with the agar-dilution method. Also, the clindamycin-resistant and susceptible phenotypes of *S. aureus* were successfully discriminated.[79] This tool, used to diagnose BSI, was widely described for its important role in clinical microbiology, since each hour of delay causes 7.6% decrease in survival for septic shock patients, within the first 6 h.[80]

Since cases of ventilator-associated pneumonia (VAP) in patients mechanically ventilated in the intensive care unit (ICU) are currently becoming recurrent, the Accelerate system was evaluated as a rapid diagnosis assay, showing capacity to detect risk of VAP in recovered patients, before the clinical signs were visible. Through a microbiological surveillance program, BAL samples were analyzed, and the automated microscopy improved the antimicrobial stewardship. The quicker diagnosis allowed to initiate an adequate antibiotic therapy, decreasing the suboptimal or inadequate use of broad-spectrum therapy instead of guided de-escalation to specific therapy. Moreover, the time reduction for identification and AST was approximately 5 h, compared to 50 ± 7 h for clinical cultures, with 100% sensitivity and 97% specificity for high-risk pneumonia organisms.[81]

Another noteworthy microfluidic image-based technology is the single-cell morphological analysis (SCMA). It uses a microfluidic agarose channel, composed of a main inlet tube which is divided in six microfluidic channels. The antibiotic diffuses to the agar-trapped cells and the bacterial growth is then monitored hourly using a microscope associated to a true-color CCD camera. Subsequently, the images are transformed into digital data and are processed using algorithms to achieve the antibiotic MIC value. This tool was evaluated for three reference strains, *E. coli* ATCC 25922, *S. aureus* ATCC 29213, and *P. aeruginosa* ATCC 27853 using antibiotics like amikacin, norfloxacin, tetracycline, and gentamicin. The MIC values were assessed in accordance with the CLSI results, obtained through the microdilution assay reference method.[82] Another study used clinical samples and a different version of the MAC chip, now in a 96-well format, to achieve high-throughput testing. The 189 clinical samples included extended-spectrum β-lactamase–positive *E. coli* and *Klebsiella pneumoniae*, imipenem-resistant *P. aeruginosa*, MRSA and vancomycin-resistant enterococci (VRE); the AST results were provided in less than 4 h with 91.5% categorical agreement, 6.5% minor, 2.6% major and 1.5% very major discrepancies. Although it rapidly achieved AST results, the main weakness of this tool remains the lack of an integrated system of identification.[83]

The Bacterial cytological profiling (BCP) is another image-based tool, that measures several different cellular parameters: changes in cell length, width, permeability, chromosome number, compactness and shape, using fluorescent dyes and a microscope. The parameters are determined by the effects of the antibiotic treatment on the cells. Compared to other tools that rely only on cell lysis information, the BCP assay is able to evaluate the single effects caused by each antibiotic, allowing to estimate a fit combination for a synergic treatment.[84] This approach can be useful in cases of infections caused by multidrug-resistant pathogens, providing rapid identification of an effective therapy.

### 3.2 Non-Imaging-Based Technologies

Several relevant tools using non-imaging approaches were developed for AST, which typically detect a specific physical property. Among these, the BacterioScan is an electro-optical based technology using laser light scattering (FLLS). It measures the angular variation in the intensity of light scattered by a laser beam that passes through a bacterial sample. The angular variation depends on the number and size of bacterial cells in suspension, allowing to detect very low values of optical density (OD) and to measure bacterial growth. Multiple measurements (every 3 min) are done and the signals, captured by a CMOS 2-dimensional camera sensor, are processed and a density value is generated. The instrument performs reliably down to a minimum density of 10^3^ CFU mL^−1^, which is commonly considered a diagnostic threshold for bacteriuria. For this reason, it was applied to urine analysis, although it cannot recognize a polymicrobial infection. The BacterioScan model 216 tabletop can process until 16 samples simultaneously[85] and was evaluated using strains of *S. aureus*, *E. coli*, and *P. aeruginosa* and different antimicrobials; a close agreement to the conventional tests used in clinical microbiology laboratories was demonstrated. The overall time of the assay, for 95% of the organisms tested, was approximately 10 h. The minimum time of inhibition was registered for *S. aureus* (32 min) and the maximum time for *P. aeruginosa* (16 h).[85]

The Lifescale instrument measures the antibiotic effects on a bacterial population by quantifying bacterial cells before and after treatment. It measures the individual cell mass and the total number of cells in a specific volume in the cell flow through a microcantilever placed inside a microfluidic channel, retrieving a MIC value.[86] The instrument recently received the CE Mark as able to perform AST directly on blood cultures in about 3 h.

### 3.3 Biochemical Methods

Several biosensors can identify and detect bacterial growth through biochemical flags from cells, such as quantitative changes in 16S rRNA,[87–89] NADH [90,91] and FADH, changes in pH,[92] or emission of light caused by gene insertion in DNA.[93]

Genefluidics developed an electrochemical-based tool that performs both identification and AST. It relies on sandwich hybridization of specific capture and detector probes of bacterial 16S rRNA. The capture and detection at the sensor surface is followed by electrochemical signal amplification with an enzyme tag, which transduces the molecular hybridization events into quantitative electrical signals. It was applied directly on viable pathogens in urine samples.[87,88] The biosensor was evaluated on reference strains of *E. coli*, *P. aeruginosa*, and *Enterococcus faecalis* at 20-min interval. The signal increase obtained with the biosensor was proportional to the cell number obtained through quantitative plating in MHA.[89] This tool was also evaluated directly in urine samples by measuring the levels of 16S rRNA within 3.5 h, in the presence and absence of antibiotics routinely used for UTIs. The overall agreement with the standard AST was 94%.[89]

Another interesting approach uses the “stochastic confinement” of individual bacteria in nanoliter droplets (nanodroplets), using a microfluidic system. Bacteria are placed into nanoliter plugs, which accelerate the detection of molecules, flags of active cellular metabolism.[90] The changes in these flags are usually registered as changes in the fluorescence intensity, which are then correlated with the efficacy of the antibiotic treatment.[90] The stationary nanoliter droplet array (SNDA)–AST system, a tool based on the nanodroplet modification, combines the Resazurin assay on a nanoliter well array containing lyophilized antibiotics. Briefly, Resazurin is reduced by electron acceptors of cellular metabolic activity such as NADH and FADH, forming Resofurin that emits fluorescence. Since only viable cells produce NADH and FADH, fluorescence emission can be correlated with the efficacy of the antibiotic treatment.[90] This method performs AST directly on urine samples, using a fast-multi-step protocol. The array consists of two rows of 8-nL wells connected by a delivery channel through which the clinical sample is injected, adding 10% Resazurin and FC-40 oil, to isolate the sample inside the wells. The platform consists of 200 wells of standard dimension to allow the trapping of an average 4CFU/well, which correspond to 5 × 10^5^ CFU mL^−1^, to improve the clinical translatability and interpretability, using standard breakpoints established by EUCAST and CLSI. As the clinical sample flows inside the antibiotic-containing wells, the changes in fluorescence are proportional to the number of bacteria in the well, allowing to measure the efficacy of the antibiotic treatment in about 6 h.[91]

A new rapid bacterial identification and AST uses bacteriophages to recognize, bind and invade specific bacteria. Their straight-forward manipulation, production and low cost allowed their use in phage-therapy[94] and more recently as rapid tools for identification/AST tests. Recombinant bacteriophages carrying the luciferase gene were developed using synthetic biology techniques by GeneWEAVE and designated Smarticles. Once they infect the specific bacterial-host, luciferase gene expression is triggered. The signal (light) produced by luciferase-associated enzymatic reactions is quantified and correlated to the number of cells in a sample.[20,93] Other phage-based tests are already available for diagnosis/treatment, such as the FDA-cleared KeyPath MRSA/MSSA Blood Culture Test, which detects the presence of *S. aureus* directly on blood cultures. The test also discriminates between MRSA and MSSA using Cefoxitin in the assay; MRSA grow in the presence of cefoxitin, resulting in signal emission, in contrast to MSSA. A recent evaluation study reported 91.8% sensitivity and 98.3% specificity for detection of *S. aureus*.[95]

### 3.4 Sequencing Technologies

High-throughput sequencing, or next-generation sequencing (NGS), is by itself the subject of several reviews, due to the widely different sequencing technologies currently available in numerous commercial platforms. Each one has its own pros and cons regarding read length (from 25 bp to 10 kb), throughput and time-per-run, dominant error type (e.g., indel, substitution and deletion), overall error rate[96,97] and of course, cost. Its successful application in the microbiology field is due to the ability to rapidly sequence entire bacterial genomes and analyze the large amount of data obtained with bioinformatic tools that detect previously described resistance determinants. Although being a high-resolution technique (single nucleotide), the associated high cost, complex workflow, need for quality control and interfering contamination events, render this technology still weak for daily use in clinical microbiology. However, several clinical microbiology laboratories already use it for rapid bacteria identification by 16S–23S rRNA sequencing, for tracking the source of infection outbreaks, for surveillance of pathogens or for other applications.[98,99] Regrettably, in the case of plasmid-mediated outbreaks, the direct repeats and insertions in the plasmids are often omitted from contigs, highlighting the need to apply alterations to the protocols.[100] Also, for the detection of novel resistance genes, uncharacterized mechanisms of resistance or altered expression of resistance genes (e.g., encoding efflux pumps or some oxacillinase genes) this technology is not useful. The possibility to guide the clinical decision, based on NGS information, is still under evaluation.[100] In one of these evaluations, the genomes of 200 bacterial isolates of *Salmonella typhimurium, E. coli, E. faecalis*, *and E. faecium*, were compared and showed a high concordance (99.74%) with phenotypic susceptibility tests.[101]

## 4 Future Technologies for Bacterial Identification and Antibiotic Profiling

Several technologies based on physical, biochemical, imaging or metabolomic approaches are emerging as rapid ID/AST alternatives, promising to revolutionize clinical diagnostics (Table 3). Their future use, as routine assays in clinical laboratories, looks promising but additional efforts in research and development are needed, as well as clinical validation and commercial viability assessment.

### 4.1 Electronic Nose

Increasing attention is being paid to electronic nose (E-nose) devices. E-noses do not detect single chemical components in a mixture, but recognize chemical fingerprint patterns through an array of semi-selective sensors for volatile organic compounds (VOCs). Several types of sensors, such as conducting-polymers and metal oxide semi-conductors are frequently used.[102]

Several versions of E-noses are under development or test in pilot evaluation studies for rapid diagnostic of infectious diseases. In the context of clinical microbiology, E-noses analyze complex VOCs mixtures produced and emitted by micro-organisms. Since these complex mixtures are highly informative, the assay has high potential to differentiate among bacterial species (Figure 3D).[103,104] The method also gives rapid feedback on the samples analyzed, and is non-invasive if testing directly breath or urine.[102,105] However, the incapacity to identify and quantify each chemical species in the usually complex VOCs mixtures, can be regarded as a weakness.[106–108] Gas chromatography, followed by mass spectrometry (GC-MS), is the gold standard technology able to close this gap.[109] The reason why this powerful technology did not emerge as routine for clinical diagnosis is its cost and slow operation, associated with expensive analytical equipment and expert operators.[110,111] More recent technologies with higher sensitivity were developed based on Ion-mobility spectrometry (IMS). An example is the hybrid technology (GC-IMS): the GC component separates the complex chemical mixture and the IMS component detects them with extreme sensitivity.[109]

In general, E-noses have been used to identify human-exhaled VOCs profiles, for the detection of respiratory infections. The Bloodhound (Scensive Ltd) device, based on conducting polymer arrays, was used for in vitro detection of *M. tuberculosis* with an accuracy of 100%.[112] The Cyranose (Smiths Detection) was used for the detection of *S. aureus, S. pneumoniae, Haemophilus influenzae*, *and P. aeruginosa* in the upper respiratory tract and was able to distinguish between control and positive samples.[113] In vivo studies, it discriminated the VOCs of exhaled breath from patients infected by *M. tuberculosis,* from control VOCs, with 72% specificity and 84% sensitivity.[114] Its use was extended to diagnosis of VAP with good results[115–117] if compared with another device, the DiagNose (C-it, Zutphen) which lacked sensitivity and specificity.[118] It also discriminated the VOCs from patients with pneumonia, from the VOCs of healthy controls, with 100% accuracy.[119] Finally, the same device allowed to identify the bacterial species in 72% of patients affected with sinusitis.[120]

A prospective cross sectional proof-of-concept study was performed for the GC-IMS E-nose in the analysis of VOCs from exhaled breath from patients and to distinguish bacterial from viral respiratory tract infections. The commercially available Breathspec GC-IMS (IMSPEX, UK) device was compared with traditional assays (multiplex RT-PCR, pathogen culture from sputum, bronchial washings or blood, chest X-ray and C reactive protein) results, chosen based on the patients symptoms. To eliminate the VOCs background, an air sample was collected immediately after the patient’s breath sample and each result was achieved in 10 min. The GC-IMS assay showed a sensitivity of 62% and 80% specificity. Despite promising, these results should be carefully interpreted, since the study showed several limitations, such as the lack of a diagnostic algorithm validated in an external cohort and the exploratory nature of the study, which analyzed a small sample size of patients with diseases that are known to affect the VOC profile.[109]

Another IMS instrument, the ChemPro 100i (Environics Inc.) was evaluated for discrimination of relevant skin and soft tissue infection (SSTI) pathogens (*S. aureus, P. aeruginosa, Enterococcus, E. coli* and *Clostridium perfringens*) from culture plates; the assay showed an accuracy of 78%, in comparison with the MALDI-TOF assay. Remarkably, it differentiated MRSA from MSSA with 83% sensitivity, 100% specificity, and 91% overall accuracy. Although the number of strains evaluated was low (12 MRSA and 11 MSSA)[121] this opens the possibility for future development as an AST technology. The same device was tested in urine samples from UTIs, and showed 95% sensitivity and 96% specificity in comparison with the reference method (urine cultures), allowing a high discriminatory power with *E. coli*, *Klebisella* spp. and a poorer discrimination and misclassification with *Staphylococcus saprophyticus* and *E. faecalis*.[122]

Few studies exist for the analysis of sepsis samples using E-noses; reference strains *E. coli* ATCC 35218, *P. aeruginosa* ATCC 27853, *S. aureus* ATCC 29213 and *E. faecalis* ATCC 29212, were inoculated in blood culture bottles, with and without supplementation with human blood and were successfully detected.[123] The same approach was also used to discriminate gram-positive and gram-negative strains in blood cultures.[124]

### 4.2 Imaging-Based Technologies

Imaging-based technologies are also among future applications. A miniaturized, single-cell imaging tool was recently developed, the fASTest device, that allows rapid AST. It consists of a microfluidic chip with two rows of 2000 cell traps of dimension 1.25 × 1.25 × 50 μm. One of the rows with trapped bacteria receives culture medium without antibiotic (representing the reference population) and the other row receives medium with antibiotic (treated population). By comparing the average growth rate of the treated population to the reference population, the system detects growth changes as fast as the biological response to the antibiotic. By measuring single cells dividing, it monitors the real-time response to an antibiotic.[125] This tool was evaluated directly on urine clinical samples with bacterial loads of 10^4^ CFU mL^−1^, the lower range for clinically relevant UTIs. The samples were loaded in 5 min and the test was performed with about 100 bacteria cells. The use of clinical samples is possible due to the small size of the bacterial traps that prevent eukaryotic cells to pass. The diagnostic tool could also detect the resistance phenotype to nine different antibiotics used for UTIs treatment, in clinical uropathogenic *E. coli* (UPEC) isolates, in less than 10 min. Ciprofloxacin resistance was detected in less than 30 min, considering the sample loading time, for 24 resistant strains and 25 susceptible strains that were grouped in agreement with gold standard disk diffusion measurements. Moreover, this technology showed the cell-shape, the different division steps (growth rates) and the different phenotypes of resistance, being able to detect polymicrobial infections or to discriminate between pathogens and contaminants.[125]

### 4.3 Nuclear Magnetic Resonance (NMR) and Raman Spectroscopy

NMR has been used to investigate the intra and extracellular bacteria composition,[126–130] and cellular metabolic pathways.[131–133] Using the extracellular metabolomic approach, NMR detects the uptake and excretion of nutrients of several bacteria. These flows represent specific metabolic footprints, applicable as bacterial identification assay as shown for patients with UTIs infections[134–136] and as AST technology.

Recently, the identification of six bacterial species (*E. coli, P. aeruginosa, Proteus mirabilis, E. faecalis, S. aureus*, *and S. saprophyticus*), frequently responsible for UTIs, was successfully achieved. Bacteria were distinguished through the production levels of several metabolites, that differed with the bacterial species. A higher amount of acetate, formate and succinate were found in *E. coli and P. mirabilis* samples, while higher amounts of glucose and serine were found in *P. aeruginosa*, *S. aureus*, *and E. faecalis* samples. These different production patterns demonstrate the usefulness of this approach as a future microbial identification method.[137] The levels of succinic acid, acetic acid, ethanol, and threonine, were evaluated for *E. coli* ATCC 25922 strain, in the presence of several concentrations of gentamicin. The level of threonine increased as the MIC of gentamicin was reached (this amino acid was not consumed due to the lack of bacterial growth) and decreased for metabolically active bacteria (below gentamicin MIC),[138] opening future uses for this approach in AST.

Using Raman spectroscopy, AST was achieved in less than 2 h for MSSA and *E. coli* reference strains and for clinical samples of *A. baumannii*, *K. pneumoniae, E. coli*, and MRSA, including VISA strains. The intensity of specific biomarkers in surface-enhanced Raman scattering (SERS) spectra of the bacteria was proportional to the antibiotic effectiveness, decreased for susceptible strains compared to resistant strains. The MIC values of each strain were in agreement with the ones obtained with traditional methods (agar dilution). Moreover, the MSSA reference strain was discriminated from the MRSA clinical isolates, using oxacillin treatment and, in the same way, *E. coli* reference strain was discriminated from the imipenem-resistant *E. coli* clinical isolates, using imipenem treatment, within 2 h. These results emphasize a future use for this technology in rapid AST.[139]

### 4.4 Other Future Technologies

Other technologies could represent valid alternatives for rapid diagnostic tools. Flow citometry assays detect viable bacteria using fluorescent dyes, capable to bind and detect nucleic acids in permeated (damaged) cells, but not in undamaged viable bacteria, allowing to evaluate the effect of antimicrobial therapies.[140,141] The isothermal microcalorimetric assay allows the measurement of heat, signal of active metabolism, in growing bacterial cells.[142,143] The magnetic bead spin assay uses antibody-coated magnetic beads that bind bacteria. The frequency of rotation of the beads when a magnetic field is applied, changes as a function of bacterial growth.[144] Finally, the impedance measurement assay evaluates the changes in emission of electrical signals by bacterial cells captured inside a microchip, in the presence and absence of antibiotic treatment.[145]

## 5 Conclusions

One of the ways to combat the spread of antimicrobial resistance is to work towards the development of accurate diagnostic technologies, which ideally should simultaneously perform the identification of the pathogen agent and the antibiotic susceptibility profiling in a second-to-minute timeframe. Such approach would allow the virtually immediate prescription of the most adequate antibiotic therapy.

The current clinical diagnostic technologies, albeit solid, easy to use and in some cases low cost, are typically time-consuming. It is anticipated that several emerging and new technologies described herein will represent the backbone of future routine microbiology assays. Their higher resolution power, ability to directly detect infection on patient samples, and the celerity to perform identification and antimicrobial susceptibility profiling are strengths over the current protocols. The technological advances in molecular-based approaches and sequencing tools, as well as on the understanding of metabolic biomarkers or profiles with high discriminatory power between pathogens, can act together to promote the efficacy of the diagnostic tools. On the other hand, the miniaturization of sensing devices, for example through the combination of microfluidics and optical tools, can promote the development of portable, user-friendly devices to be used at the point-of-care. Some critical aspects that will need more attention in the future are the adequacy of non-invasive methodologies, and also the adaptation of protocols to include slow growing pathogens such as *M. tuberculosis,* or fastidious and anaerobic microorganisms. Ideally, the possibility to distinguish between resistance, tolerance, and persistence to antibiotic treatments would also represent an important breakthrough.

## Figures and Tables

**Table 1:** Selected examples of current technologies for pathogen identification (ID) and antimicrobial susceptibility testing (AST).

Currently used technologies	ID & AST technologies	Example of assay manufacturer	Summary of method	Time of AST	Directly on patient sample	Real MIC	POP or CA	FDA or CE approved	Costs of equipment and test supplies	Automatic or manual	References
Culture-based	Agar diluition assay	Oxoid	Bacteria inoculated on agar plates with different concentrations of antibiotics	16–24 h	No	Yes/no	CA	FDA	+	M	14
	Disk diffusion	Oxoid	Bacteria inoculated on agar plates with single antibiotic disks	16–24 h	No	Yes/no	CA	FDA	+	M	14,15
	Gradient test	bioMérieux	Bacteria inoculated on agar plates with graded antibiotic concentration strips	16–24 h	No	Yes	CA	FDA	+	M	14,15
	Broth dilution assay	Oxoid	Bacteria inoculated in liquid media with different antibiotics to monitor growth	12–24 h	No	Yes	CA	FDA	+	M	14
	MicroScan WalkAway	Beckman Coulter	Measure bacterial growth in the presence of antibiotics by recording bacterial turbidity using a photometer	4.5–18 h	No	Yes	CA	FDA	$$/++	A	4,146
	Vitek bioMérieux	bioMérieux	Measure bacterial growth in the presence of antibiotics by recording bacterial turbidity using a photometer	6–11 h	No	Yes	CA	FDA	$$/++	A	20
	BD phoenix	Becton Dickinson	Record bacterial growth in the presence of antibiotics by recording bacterial turbidity and colorimetric changes	9–15 h	No	Yes	CA	FDA	$$/++	A	20
	Sensititre	Thermo Fisher Scientific	Record bacterial growth with antibiotics by measuring fluorescence	18–24 h	No	Yes	CA	FDA	$$/++	A	14
Molecular based	LPA line probe assay	Autoimmun Diagnostika (AID)	PCR followed by hybridization with DNA probes present on the nitrocellulose strip followed by signal detection of hybridized biotinylated PCR amplicons	>6 h	Y(Urine)/N	No	CA	CE	$/+	M	42,41
	Gene xpert system	Cepheid	DNA amplification using qRT-PCR to detect methicillin resistance or susceptibility (MRSA/MSSA) in positive blood culture	>1 h	No	No	CA	FDA	$$$/+++	A/M	29
	Septifast	Roche	Real-time PCR followed by highly specific melting point analysis using specific hybridization probes (FRET)	6 h	Y(Blood)	No	CA	CE	$$/+++	A	29
	FilmArray	BioFire	Double PCR reaction in a row: multiplex PCR followed by nested PCR and amplicon melting analysis	1 h	Y(Blood)	No	CA	FDA and CE	$$/+++	A	47,48
	Verigene	Nanosphere	Microarray of oligonucleotide probes, designed to specifically bind several DNA sequences of different target pathogens	>2 h	No	No	CA	FDA	$$/+++	A	52,53
Spectrometry	MALDI TOF-MS	Bruker Daltonics	Identification of molecules based on their time of flight though a vacuum tube after laser irradiation of a matrix that is co-crystallized with sample, generating a spectrum that is after compared with a reference database	<5 h	No	No	CA	FDA	$$$/+	A	60
Molecular detection & spectrometry-based	Iridica	Ibis Biosciences	PCR/electrospray ionization mass spectrometry (ESI-MS)	<6 h	Y (blood)	No	CA	CE	$$$$/+++	A	73,75

**Table 2:** Selected examples of emerging technologies for pathogen identification (ID) and antimicrobial susceptibility testing (AST).

ID & AST technologies	Example of assay manufacturer or technology	Assay manufacturer	Summary of method	Time of AST	Direct on patient sample	Real MIC	POP or CA	FDA or CE approved	Automatic or manual	References
Molecular & imaging-based	Accelerate pheno system	Accelerate Diagnostics	Multiplexed automated digital microscopy (MADM)	<6 h	Yes (positive blood-culture) and (BAL)	Yes	CA	FDA	A	20,67,69,70,77, 79,81,83,87
Imaging-based	oCelloscope	Philips BioCell	Digital time-lapse microscopy scanning population of bacterial cells	1–4 h	Yes (urine) (positive blood-culture)	Yes	POP	No	A	65,66
	Bacterial cytologial profiling		Fluorescence microscopy to analyze a multitude of parameters to discriminate resistant from susceptible strains	<2 h	No	Yes	POP	No	A	84
Imaging-based & microfluidics	SCMA		Single-cell morphological analysis (SCMA) performed in microfluidic agarose channels (MAC) system	<4 h	No	Yes	POP	No	A	82,83
Non imaging-based & microfluidics (lab on chip)	Nanodroplets/nanoliter arrays		Measurement of the metabolically active bacteria	<6 h	Yes (urine)	Yes	POP	No	A	90,91
	UtiMax™	GeneFluidics	Electrochemical measurement of bacterial 16S rRNA	<4 h	Yes (urine)	Yes	CA	CE	A	87–89
	pH change		Microfluidic pH sensor detect metabolic products by bacteria growth	<3 h	No	Yes	na		A	92
	LifeScale	Affinity Biosensor	Resonant mass measurement	>3 h	Yes (urine)	Yes	CA	CE	A	86
Non imaging-based	BacterioScan	BacterioScan, Inc.	The laser light scattering (FLLS) determines the number and size of bacterial cells suspended in a solution	3–10 h	Yes (urine)	Yes	CA	No	A	85,147
Molecular and biochemical-based	Next-generation sequencing and whole generation sequencing		Sequencing entire bacterial genomes or RNA	>10	No	No	CA	na	A	99–101,148
	Smarticles	Roche Diagnostics	Bacteriophages carrying luciferase gene- infect bacteria producing detectable light signals	ND	ND	ND	POP	No	ND	93
	KeyPath MSSA/MRSA blood culture test	Microphage	Detection of phage antigens as a surrogate for the presence of bacteria in the sample	<5 h	Yes (positive blood-culture)	No	CA	FDA	M	95

CA, clinically approved; MIC, minimum inhibitory concentration; POP, proof of principle.

**Table 3:** Selected examples of future technologies for pathogen identification (ID) and antimicrobial susceptibility testing (AST).

Technologies	Summary of method	Time of AST	Direct on patient sample	Real MIC	POP or CA	Automatic or manual	References
E-nose	Detection of VOCs as an electronic aroma signature to identify bacteria and recently to discriminate MRSA from MSSA	NA	Yes (urine, breath, positive blood culture)	No	CA	A	[114,119,121–124]
Flow cytometry	Follow the viability of microorganisms, after exposure to antibiotics using dyes that do not permeate the cell walls of healthy bacteria	2–3 h	No	Yes	POP	A	[140,141]
IMC (isothermal microcalorimetry)	Measure the heat as signature of growing cells	3–14 h	Yes (urine)	Yes	POP	A	[142,143]
Magnetic bead spin	Changes in spin of magnetic beads in a magnetic field as a function of the number of bacteria bound	<5 h	No	Yes	POP	A	[144]
NMR spectroscopy	Analysis of the bacteria metabolome, using it to identify different bacteria and its antimicrobial susceptibility phenotype.	<6 h	No	Yes	POP	A	[137,138]
fASTest	Direct single-cell imaging using microfluidic chip	<30 min	Yes (urine)	Yes	POP	A	[125]
Impedance measurement	Measure the electrical response from target bacteria in the presence and absence of antibiotics	<90 min	Yes (blood) (urine)	No	POP	A	[145]
Infrared spectroscopy	Discriminate the strains on the basis of their infrared spectra	ND	No	No	POP	A	[149]
Surface-enhanced raman scattering (SERS)	Measure the intensity of specific bacteria biomarkers using Raman scattering (SERS) spectra	2 h	No	Yes	POP	A	[139]

CA, clinically approved; MIC, minimum inhibitory concentration; POP, proof of principle.

## Abbreviations

AMR: antimicrobial resistance
AST: antimicrobial susceptibility testing
BAL: bronchoalveolar lavage
BCID: blood culture identification
BCP: bacterial cytological profiling
BSI: bloodstream infections
CFU: colony-forming unit
CLSI: Clinical and Laboratory Standards Institute
ECDC: European Centre for Disease Prevention and Control
EDTA: ethylene-diamine-tetraacetic acid
EMEA: European Medicines Agency
ESBL: extended-spectrum beta-lactamase
EUCAST: European Committee on Antimicrobial Susceptibility Testing
FADH: flavin adenine dinucleotide reduced
FDA: Food and Drug Administration
FISH: fluorescent in situ hybridized
FLLS: forward laser light scattering
FRET: fluorescent resonance energy transfer
GC: gas chromatography
HRM: high-resolution melting
hVISA: heteroresistant vancomycin-intermediate *Staphylococcus aureus*
ICU: intensive care unit
IMS: ion mobility spectrometry
MAC: microfluidic agarose channel
MADM: multiplexed automated digital microscopy
MALDI-TOF MS: matrix-assisted laser desorption ionization-time of flight mass spectrometry
MDR: multidrug-resistant
MHA: Mueller-Hinton agar
MIC: minimum inhibition concentration
MRSA: methicillin-resistant *Staphylococcus aureus*
MSSA: methicillin susceptible *Staphylococcus aureus*
NADH: nicotinamide adenine dinucleotide reduced
NMR: nuclear magnetic resonance
PCR: polymerase chain reaction
PCR/ESI-MS: polymerase chain reaction/electrospray-mass spectrometry
qRT-PCR: real-time PCR
SCMA: single-cell morphological analysis
SERS: surface-enhanced Raman scattering
UTI: urinary tract infections
VAP: ventilator-associated pneumonia
VISA: vancomycin-intermediate *Staphylococcus aureus*
VSSA: vancomycin-susceptible *Staphylococcus aureus*
WHO: World Health Organization
